# Missense mutation at *CLDN8* associated with a high plasma interferon gamma-inducible protein 10 level in methadone-maintained patients with urine test positive for morphine

**DOI:** 10.1371/journal.pone.0187639

**Published:** 2017-11-16

**Authors:** Tung-Hsia Liu, Ren-Hua Chung, Sheng-Chang Wang, Chiu-Ping Fang, Hsiao-Hui Tsou, Chia-Lung Shih, Hsiang-Wei Kuo, Yun Wang, Yu-Li Liu

**Affiliations:** 1 Center for Neuropsychiatric Research, National Health Research Institutes, Miaoli, Taiwan; 2 Division of Biostatistics and Bioinformatics, Institute of Population Health Sciences, National Health Research Institutes, Miaoli, Taiwan; 3 Graduate Institute of Biostatistics, College of Public Health, China Medical University, Taichung, Taiwan; 4 Graduate Institute of Clinical Medical Science, China Medical University, Taichung, Taiwan; China Medical University, TAIWAN

## Abstract

We previously reported a high plasma chemokine interferon gamma-inducible protein 10 (IP-10) level and prolonged electrocardiography QT-interval in methadone maintenance treatment (MMT) patients with HIV or HCV infection. The purpose of this study was to evaluate the genetic association of high plasma IP-10 level in the MMT patients. The gene-based and pathway-based association analyses were conducted using a genome-wide association study dataset in 344 MMT patients for identifying genes and pathways associated with plasma IP-10 level. We found that plasma IP-10 level was significantly associated with a pathway in the tight junction (*P* = 1.01x10^-5^), where the claudin 8 (*CLDN8*) gene had the most significant association (*P* = 6.8x10^-5^). A functional single nucleotide polymorphism (SNP) rs686364 at exon 1 of *CLDN8* showed strong association with plasma IP-10 levels, in the MMT subjects with positive urine test for morphine (dominant model, *P* = 0.00004). The minor allele type carriers had higher plasma IP-10 levels than the major allele type carriers. Our data support that the tight junction protein claudin 8 exon 1 is a predictor for the plasma levels of IP-10 in MMT patients with urine test positive for morphine.

## Introduction

Methadone is a synthetic opioid used for the treatment of heroin dependence [1–3]. We previously reported a high prevalence of hepatitis C viral (HCV, 95%) and human immunodeficiency virus (HIV, 23%) infection in a methadone maintenance treatment (MMT) population in Taiwan [4]. These HCV or HIV patients showed an increase in plasma levels of chemokine interferon gamma-inducible protein 10 (IP-10; also called chemokine CXC motif ligand 10; CXCL10) [5, 6]. IP-10 is a chemoattractant of proinflammatory mediator for T cell activation and adhesion to endothelial cells [7, 8]. High plasma IP-10 level correlated with the prolonged electrocardiogram (ECG) QTc interval, a potentially lethal side effect of methadone [9, 10], in these patients [5]. High plasma IP-10 level is also associated with other inflammatory diseases. For example, the severity of clinical symptoms of lymphoproliferative disorder [11], systemic lupus erythematosus [12], essential hypertension [13], type II diabetes [14], Kawasaki disease [15] and HIV infection [16] correlated well with plasma IP-10 level in patients. These data suggested that IP-10 may be a risk factor and a potential therapeutic target for inflammation [6]. However, there is no genetic marker to predict the high levels of plasma IP-10 in patients.

In the current study, we used the gene-based and pathway-based association analyses, methods commonly applied as a secondary analysis strategy in genome-wide association studies, to identify a candidate gene claudin 8 (*CLDN8*) in the pathway of tight junction interactions associated with the plasma IP-10 levels. CLDN8 is mainly expressed in endothelial cells which exert physiological functions as tight junctions in kidney and gastrointestinal tract [17–22]. We found that *CLDN8* gene is involved in the pathogenesis of high IP-10 level, and an amino acid change in *CLDN8* was associated with high plasma levels of IP-10.

## Materials and methods

### Subject

The recruitments were approved by the institutional review boards of the National Health Research Institutes (Miaoli County, Taiwan) (Permit Number: EC0970504) and the 6 participating hospitals. Written informed consents were obtained from all participants. The projects had also been registered with the National Institutes of Health Clinical Trial database (https://clinicaltrials.gov/ct2/show/results/NCT01059747). The inclusion criteria included an age of 18 years or above, receipt of MMT for at least three months with regular attendance for the past seven days, and a methadone dosage adjustment of no more than 10 mg in the past seven days. Exclusion criteria included co-morbidity with physical or mental disorders requiring immediate treatment and pregnancy. A total of 344 MMT patients were recruited from 6 hospitals in the first study [23].

### Clinical assessment

The clinical characteristics and methadone treatment courses, including the dose and treatment duration, and the treatment adherence over the previous week, were obtained from patients’ medical records. All the assessments including plasma IP-10 levels were reported in our previous study [5]. Chemokines IP-10 levels were determined using the Milliplex® MAP human cytokine/chemokine kit (Millipore, Billerica, MA).

### Genome-wide SNP genotyping

Genomic DNA was extracted from blood using the Puregene DNA Isolation Kit (Gentra Systems). Each individual was genotyped using the Axiom Genome-wide CHB 1 Array, which was population-optimized to have a better genomic coverage of common alleles (MAF>5%) of the Han Chinese genome. Genotype calling was performed using Genotyping Console 4.0 with default parameters (http://www.affymetrix.com). Three hundred forty four samples/615,216 SNPs passed the quality control and were used for the analysis [23].

### Urine morphine test

Urine specimens were collected prior to the administration of methadone on the recruiting day. The morphine screen test was performed via a kinetic interaction of microparticles (KIMS) on an Integra 800 device (Roche Diagnostics, Basel, Switzerland). The test was used as a surrogate measurement for the methadone response, where the presence of morphine in urine was considered as urine morphine test positive to the MMT treatment.

### Statistical analyses

Statistical analyses were conducted using the SAS software, Version 9.4 (SAS Institute, Inc., Cary, NC) and other publicly available tools for genetic studies. Before the genetic association analyses, the plasma IP-10 data were natural log transformed and achieved the normality assumption by the Shapiro-Wilk tests using SAS. Genome-wide single-marker association statistics were calculated by PLINK [24], Version 1.07 with covariates including age, gender and body mass index (BMI). Based on the single-marker association statistics, the gene-based and pathway-based association analyses were analyzed by Knowledge-based mining system for Genome-wide Genetic studies (KGG [25, 26], Version 2.5). The gene-based and pathway-based *P*-values were calculated by the extended Simes procedure (GATES) and Hybrid set-based test (HYST), respectively, in KGG. The pathway-based association analysis method aggregated gene-based *P*-values into a pathway-based *P*-value. We also investigated the associations of individual SNPs in *CLDN8* with plasma IP-10 levels. These associations between SNPs of *CLDN8* (genotype and dominant model) and plasma IP-10 levels were tested using the SAS GLM procedure and the multiple comparisons correction for FDR using the MULTTEST procedure. The Hardy-Weinberg equilibrium tests for these SNPs were performed using HAPLOVIEW version 4.2 [27].

## Results

### MMT patient profile

Table 1 summarized the statistics of 344 MMT patients used in analyses. The average age was 38.16 ± 7.69 years old. The majority of the patients were male. Their average plasma IP-10 level was 1164.75 ± 803.73 pg/ml.

**Table 1:** Demography of the methadone maintenance treatment patients.

Variable	n	Mean	±	SD
Age (years)	344	38.16	±	7.69
Male (%)	281	(81.69%)		
BMI (kg/m^2^)	341	23.64	±	3.52
Methadone dosage (mg/day)	344	55.22	±	28.47
Addiction duration (year)	344	12.98	±	7.50
Urine morphine (+) (%)	173	(50.58%)		
IP-10, pg/ml	339	1164.75	±	803.73

BMI, Body Mass Index. SD, Standard deviation.

### The association of CLDN8 with plasma IP-10 level

The PLINK linear regression, assuming an additive model, was used to calculate the single-marker association statistics for the 615,216 SNPs. Pathway definitions from KEGG, Reactome, and BioCarta pathway databases were used in KGG to perform the gene- and pathway-based association tests. A total of 16,130 genes for gene-based tests and 1,421 pathways in pathway-based tests were used. A pathway of tight junction interactions from the Reactome pathway database significantly associated with the natural log-transformed plasma IP-10 levels (*P* = 1.01x10^-5^), with the stepdown Bonferroni corrected *P*-value of 0.0143 and FDR of 0.0143 for testing the 1,421 pathways (Table 2). Thus, the *P*-value of tight junction interaction pathway passed the genome-wide significance threshold. Amongst the genes in the tight junction interaction pathway, *CLDN8* was highly associated with plasma IP-10 level with the lowest *P*-value of 6.8x10^-5^ (Table 2). The MMT patients were further separated into two groups based on a urine morphine test. *CLDN8* gene was significantly associated with plasma IP-10 level in the urine morphine positive (*P* = 0.005), but not the urine morphine negative (*P* = 0.106) patients.

**Table 2:** The interactions of tight junction proteins and plasma IP-10 in MMT patients.

Pathway / Gene	Chromosome	Gene-based *P*-value	
		All MMT patients	MMP patients with UMP
**Tight junction interactions**		**0.00001**	**0.005**
*CLDN8*	21	**0.000068**	**0.0005**
*CLDN11*	3	**0.005**	**0.002**
*CLDN10*	13	**0.020**	0.265
*PARD3*	10	**0.024**	0.231
*INADL*	1	**0.040**	**0.003**
*CLDN14*	21	**0.041**	0.233
*CLDN20*	6	0.052	0.451
*CLDN7*	17	0.097	0.498
*MPP5*	14	0.117	0.406
*F11R*	1	0.234	0.487
*CLDN12*	7	0.235	0.065
*CLDN16*	3	0.263	0.139
*CLDN15*	7	0.320	0.748
*PARD6G*	18	0.448	0.827
*CLDN18*	3	0.608	0.602
*PARD6B*	20	0.747	0.910
*PRKCI*	3	0.750	0.371
*CLDN1*	3	0.837	0.358

UMP, urine morphine positive.

### Significant association of exon 1 SNP with plasma IP-10 levels

*CLDN8* is a gene spanning for 15,170 base pair lengths at chromosome 21q22.11 region. It has 5 SNPs rs2510527 (downstream), rs686364 (exon 1), rs2832657 (promoter), rs16986270 (promoter), and rs670864 (promoter) in the genome-wide association dataset at its genetic coding region (S1 Table). All SNPs passed the Hardy Weinberg’s equilibrium tests at the significance level of 0.05, except for rs2832657.

The exon 1 SNP rs686364 showed a significant association with plasma IP-10 level (GLM, *P* = 0.00002) using dominant model of analyses in the MMT patients (Table 3). This significance was contributed mainly from the urine morphine positive patients (GLM, *P* = 0.00004) (Table 3), but not in the negative (dominant model, GLM, *P* = 0.068) patients. The G allele type carriers had higher plasma IP-10 levels (1285.9 ± 877.1 pg/ml) than the AA genotype carriers (940.7 ± 586.9 pg/ml) in the MMT patients. The age, gender, BMI, methadone dose, addiction duration, and percentage urine morphine positive were not different between the G-allele type carriers and the AA genotype carriers on rs686364 (S2 Table).

**Table 3:** The dominant model association analyses between the SNPs of *CLDN8* and IP-10 (pg/ml) in all MMT patients and urine morphine positive MMT patients.

SNP	Dominant model	MMT patients					UMP MMT patients				
		N	Mean	±	SD	*P*-value (FDR)	N	Mean	±	SD	*P*-value (FDR)
rs2510527 (Downstream)	GG	165	1216.63	±	859.69	0.366	84	1157.89	±	702.27	0.814
	AG+AA	174	1115.54	±	745.96	(0.392)	86	1179.68	±	753.61	(0.814)
rs686364 (Exon 1)	AA	119	940.74	±	586.91	**0.00002**	55	884.22	±	587.22	**0.00004**
	AG+GG	220	1285.91	±	877.13	**(0.0001)**	115	1305.07	±	749.38	**(0.0002)**
rs2832657 (Promoter)	GG	98	995.18	±	586.22	**0.028**	47	949.21	±	558.28	**0.038**
	GT+TT	237	1242.46	±	872.47	(0.063)	122	1254.00	±	769.98	(0.063)
rs16986270 (Promoter)	AA	228	1197.00	±	850.35	0.392	108	1202.16	±	772.88	0.712
	AG+GG	111	1098.49	±	697.22	(0.392)	62	1111.00	±	640.00	(0.814)
rs670864 (Promoter)	CC	220	1104.80	±	757.00	**0.038**	112	1077.28	±	675.96	**0.023**
	AC+AA	119	1275.56	±	876.19	(0.063)	58	1345.87	±	792.00	(0.058)

SD, standard deviation. Bold form, *P*<0.05

UMP, urine morphine positive.

*P*-value and FDR in general linear model (GLM) adjusted for age, gender and BMI by nature log transformed IP-10.

## Discussion

High plasma or serum IP-10 was found in patients with lymphoproliferative disorder [11], systemic lupus erythematosus [12], HIV infection [16], or Kawasaki disease of acute vasculitis [15]. We previously also reported a high plasma IP-10 level in the MMT patients with HIV and HCV infection [5]. In the present study, we identified a pathway with genome-wide significance and pinpointed a candidate gene correlating the plasma levels of IP-10 in MMT patients. Plasma IP-10 level was strongly associated with a tight junction membrane protein. *CLDN8* [28] Our data suggest that *CLDN8* genetic variant may influence plasma IP-10 level in MMT patients.

The exon 1 SNP rs686364 is a missense genetic polymorphism encoding the amino acid number 151 in the CLDN8 protein. The A major allele type encodes the serine residue and the G allele type encodes the proline residue (S1 Fig). The major AA genotype carriers had lower plasma IP-10 levels, whereas the minor G allele type carriers had higher plasma IP-10 levels. A differential population distribution frequency of exon 1 SNP rs686364 of the A and mutant G allele type carriers was identified from 1000 Genome (https://goo.gl/2yvkfa) among the ethnic groups. As seen in S2 Fig, the Africans are 72% of G allele type carriers, the Han Chinese and East Asian have 40.7% and 44.8% G allele type carriers. The South Asian, American, and European have 23.3%, 26.51%, and 27.7% G allele type carriers. These results indicate the potential influences in different ethnic groups.

Five SNPs were located at the *CLDN8* genetic region within the MMT patients’ genomewide genotype database. The SNP rs686364 located at exon 1 encodes a missense mutation, where the A allele is translated into serine amino acid and the minor G allele is translated into proline amino acid at the claudin 8 151 protein encoding position (S151P) (S1 Fig). The AA genotype carrier had lower plasma IP-10 levels than the G allele type carrier. These data suggest that the mutation of *CLDN8* gene in the G-allele type carriers may impair the tight junction and result in the leakage of IP-10 into the blood stream. Our data also support the exon 1 SNP rs686364 as an indicator for plasma IP-10 levels.

We found that the average minor G allele frequency in this *CLDN8* SNP rs686364 is approximately 42%. The estimated allele frequencies for the G allele at the SNP are 25% in the European population and 78% in the African population (https://goo.gl/ku4Fwz). As seen in S2 Fig, the G allele has very different allele frequencies among these ethnic groups, which may have different effects on altering junction function, inflammation or MMT effectiveness among the ethnic groups.

We previously reported an elevated plasma IP-10 levels in MMT patients with HIV and HCV infection [5]. In this study, MMT patients without HIV or HCV infection also had a high plasma IP-10 level than the normal controls (S3 Table). These data suggest that the use of opioids may increase plasma IP-10 level. The average plasma IP-10 levels were not different among genotypes between urine morphine positive and morphine negative patients. Therefore, the SNP showed association with plasma IP-10 levels mainly in urine morphine positive patients could be due to the combine use of other opioid which adjusted the plasma IP-10 levels and reduce the standard deviation (SD) in urine morphine positive patients. The high IP-10 level was associated with a prolonged cardiac QT interval [5]. We demonstrated a high plasma IP-10 level in a polymorphism at CLDN8 exon1 rs686364 with G allele carrier. This SNP may serve as a biomarker for the QT prolongation side effect in MMT patients. The influences of this CLDN8 SNP for other immunological disorders warrant further replication.

Claudin 8 is an encoding protein of *CLDN8* gene and belongs to a member of tight junction strand. Previous studies have indicated that claudin 8 contributed to paracellular barrier in the distal renal tubule [29] and distal colon to prevent sodium back-leakage [30]. CLDN8 is also expressed in mammalian intestines [31], where the amino acid number 151 encoding serine to proline in human claudin 8 significantly affected the *Clostridium perfringens* enterotoxin (CPE) binding ability [32]. In this study, we demonstrated that single amino acid mutation at protein 151 position of claudin 8 altered the release of IP-10. Using NCBI bioinformatics, we found that IP-10 (https://goo.gl/zEgmZH) and CLDN8 (https://goo.gl/eydC5i) co-expressed highly in kidney, intestine, lungs, mammary gland, and prostate (S4 Table), suggesting that the CLDN8 might have more influence on the IP-10 release from these tissues.

Some limitations should be considered in this study. A larger sample will be helpful to further verify this observation. The exon 1 rs686364 on *CLDN8* tight junction association with plasma IP-10 levels is first reported in a Taiwan population, which passed the genome-wide significance threshold. This finding should be verified in other ethnic groups. Animal studies confirming that claudin 8 influences the release of IP-10 are warranted.

In summary, a tight junction interaction pathway was identified responsible for the plasma IP-10 levels using pathway-based association analyses. A candidate gene *CLDN8* showed strong associations with plasma IP-10 levels. The missense SNP rs686364 at exon 1 encoded the 151 amino acids was associated with plasma IP-10 levels, where the major AA genotype carrier had lower plasma IP-10 than the minor G allele type carriers. This association was mainly contributed from the urine morphine positive of MMT patients. Our data support a genetic marker at exon 1 of *CLDN8* has the potential to predict plasma IP-10 levels in heroin dependent patients under methadone maintenance treatment.

## Supporting information

S1 Fig The (A) *CLDN8* gene and (B) protein structure of rs686364 has missense function from a substitution of serine (Ser) to proline (Pro) at position 151.
(DOC)

S2 Fig The functional SNP rs686364 population of allele frequencies from 1000 Genome (https://www.ncbi.nlm.nih.gov/projects/SNP/snp_ref.cgi?rs=686364). SNP rs686364 encodes a missense mutation, the allele change from A allele to G allele. The abbreviation represents Han Chinese in Beijing, China (HCB), East Asian (EAS), South Asian (SAS), American (AMR), European (EUR), and African (AFR) populations. In the pie chart, A and G represents allele type and the percentage after the comma.
(DOC)

S1 Table *CLDN8* single nucleotide polymorphisms on chromosome 21 within the genome-wide genotyping database. (DOC)

S2 Table The dominant model association analyses between the rs686364 and demography of subjects removed HCV (-)/HIV (-) MMT patients. (DOC)

S3 Table Association analyses between the normal control and HIV (-)/HCV (-) in MMT patients with IP-10 (pg/ml). (DOC)

S4 Table Gene expression profiles for IP-10 and CLDN8 in tissues. (DOC)
