# Supplementary material for: Missense mutation at CLDN8 associated with a high plasma interferon gamma-inducible protein 10 level in methadone-maintained patients with urine test positive for morphine
Source: PLoS One. 2017 Nov 16;12(11):e0187639. doi: 10.1371/journal.pone.0187639 (PMC5690676; doi:10.1371/journal.pone.0187639)

**S1 Fig.** The (A) *CLDN8* gene and (B**)** protein structure of rs686364 has missense function from a substitution of serine (Ser) to proline (Pro) at position 151.


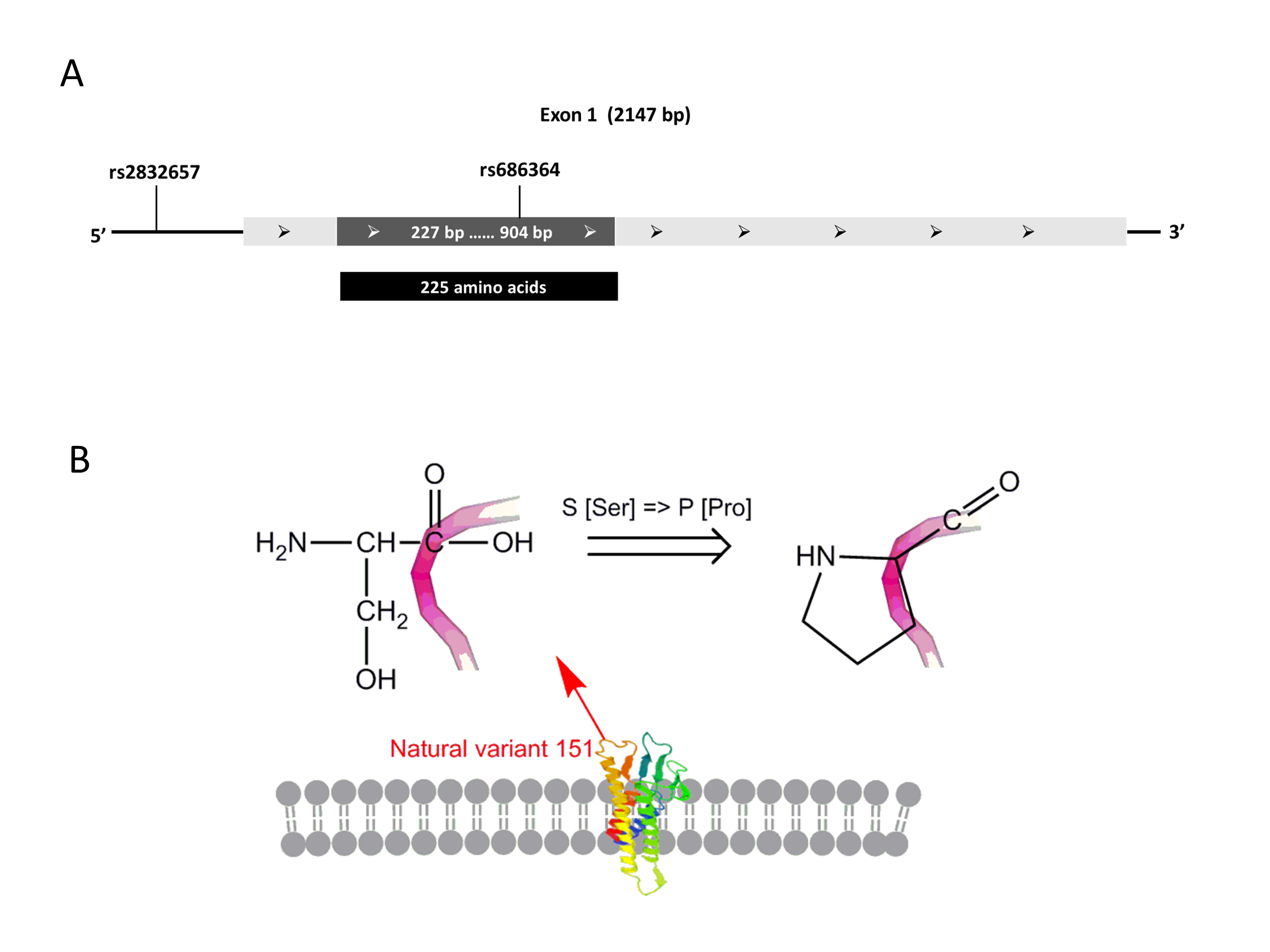

Supplement: S1 Fig — The (A) CLDN8 gene and (B) protein structure of rs686364 has missense function from a substitution of serine (Ser) to proline (Pro) at position 151. (DOC) [file pone.0187639.s001.doc]
