# Supplementary material for: Missense mutation at CLDN8 associated with a high plasma interferon gamma-inducible protein 10 level in methadone-maintained patients with urine test positive for morphine
Source: PLoS One. 2017 Nov 16;12(11):e0187639. doi: 10.1371/journal.pone.0187639 (PMC5690676; doi:10.1371/journal.pone.0187639)

**S2 Fig.** The functional SNP rs686364 population of allele frequencies from 1000 Genome (https://www.ncbi.nlm.nih.gov/projects/SNP/snp_ref.cgi?rs=686364). SNP rs686364 encodes a missense mutation, the allele change from A allele to G allele. The abbreviation represents Han Chinese in Beijing, China (HCB), East Asian (EAS), South Asian (SAS), American (AMR), European (EUR), and African (AFR) populations. In the pie chart, A and G represents allele type and the percentage after the comma.


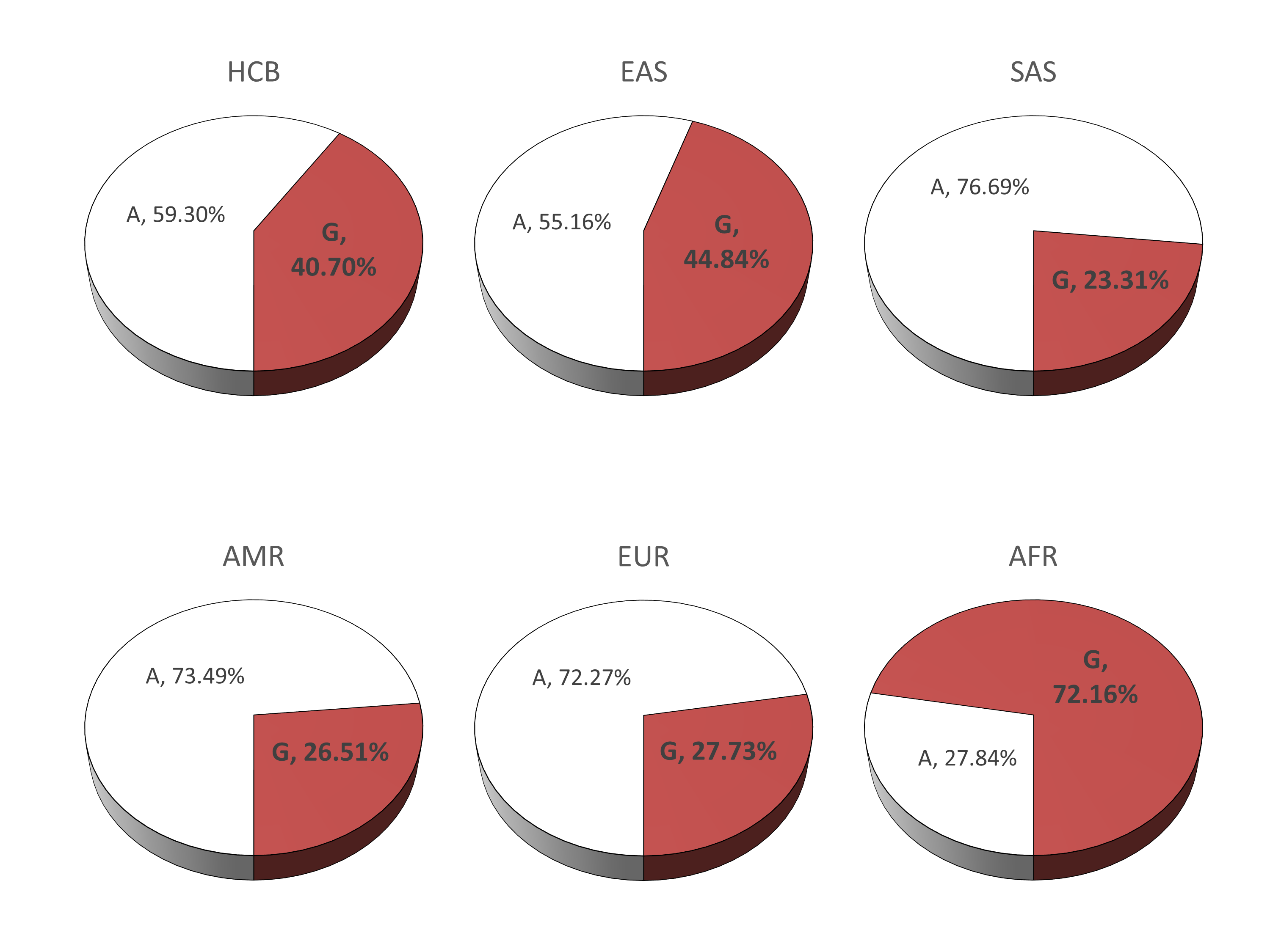

Supplement: S2 Fig — SNP rs686364 encodes a missense mutation, the allele change from A allele to G allele. The abbreviation represents Han Chinese in Beijing, China (HCB), East Asian (EAS), South Asian (SAS), American (AMR), European (EUR), and African (AFR) populations. In the pie chart, A and G represents allele type and the percentage after the comma. (DOC) [file pone.0187639.s002.doc]
