# Supplementary material for: Missense mutation at CLDN8 associated with a high plasma interferon gamma-inducible protein 10 level in methadone-maintained patients with urine test positive for morphine
Source: PLoS One. 2017 Nov 16;12(11):e0187639. doi: 10.1371/journal.pone.0187639 (PMC5690676; doi:10.1371/journal.pone.0187639)
Supplement: S1 Table — (DOC) [file pone.0187639.s003.doc]

**S1 Table. *CLDN8* single nucleotide polymorphisms on chromosome 21 within the genome-**wide genotyping database.

| SNP | Allele | Location | MAF | HWP |  |
| --- | --- | --- | --- | --- | --- |
| rs2510527 | G/A | Downstream | 0.298 | 0.814 |  |
| rs686364 | A/G | Exon 1 | 0.420 | 0.385 |  |
| rs2832657 | G/T | Promoter | 0.490 | **0.048** |  |
| rs16986270 | A/G | Promoter | 0.183 | 0.455 |  |
| rs670864 | C/A | Promoter | 0.202 | 0.392 |  |

Localization: according to the isoform of CLDN8 mRNA (NM_199328).

MAF: Minor allele frequency. HWP: *P*-value of Hardy-Weinberg equilibrium test.
