# Supplementary material for: Missense mutation at CLDN8 associated with a high plasma interferon gamma-inducible protein 10 level in methadone-maintained patients with urine test positive for morphine
Source: PLoS One. 2017 Nov 16;12(11):e0187639. doi: 10.1371/journal.pone.0187639 (PMC5690676; doi:10.1371/journal.pone.0187639)
Supplement: S2 Table — (DOC) [file pone.0187639.s004.doc]

**S2 Table. The dominant model association analyses between the rs686364 and demography of subjects** **removed HCV (-)/HIV (-) MMT patients.**

|  | Major genotype of rs686364: AA | | | |  | rs686364: AG+GG | | | |  |
| --- | --- | --- | --- | --- | --- | --- | --- | --- | --- | --- |
| Variable | N | Mean | ± | SD |  | N | Mean | ± | SD | *P*-value |
| Age (years) | 112 | 38.16 | ± | 8.10 |  | 215 | 38.27 | ± | 7.59 | 0.598 ^a^ |
| Male (%) | 94 | ( 83.93% ) | | |  | 171 | ( 79.53% ) | | | 0.336 ^b^ |
| BMI (kg/m^2^) | 111 | 23.83 | ± | 3.55 |  | 213 | 23.61 | ± | 3.57 | 0.478 ^a^ |
| Methadone dosage (mg/day) | 112 | 54.79 | ± | 28.18 |  | 215 | 56.70 | ± | 28.57 | 0.650 ^a^ |
| Addiction duration (year) | 112 | 13.76 | ± | 7.79 |  | 215 | 13.14 | ± | 7.31 | 0.520 ^a^ |
| Urine morphine (+) (%) | 53 | ( 47.75% ) | | |  | 113 | ( 52.80% ) | | | 0.387 ^b^ |
| SD, Standard deviation. | | | | | | | | | | |
| ^a^ Wilcoxon rank-sum test. ^b^ Chi-square test. | | | | | | | | | | |
