# Supplementary material for: Missense mutation at CLDN8 associated with a high plasma interferon gamma-inducible protein 10 level in methadone-maintained patients with urine test positive for morphine
Source: PLoS One. 2017 Nov 16;12(11):e0187639. doi: 10.1371/journal.pone.0187639 (PMC5690676; doi:10.1371/journal.pone.0187639)
Supplement: S3 Table — (DOC) [file pone.0187639.s005.doc]

**S3 Table. Association** analyses between the Normal control and HIV (-)/HCV (-) in MMT patients with IP-10 (pg/ml).

|  | IP-10, pg/ml | | | |  |
| --- | --- | --- | --- | --- | --- |
| Group | N | Mean | ± | SD | *P*-value |
| Normal control | 26 | 260.2 | ± | 152.66 | <0.0001 |
| HIV (-)/HCV (-) in MMT | 17 | 512.84 | ± | 315.85 |  |
| SD, Standard deviation.  Wilcoxon rank-sum test was used. | | | | | |
