# Supplementary material for: Missense mutation at CLDN8 associated with a high plasma interferon gamma-inducible protein 10 level in methadone-maintained patients with urine test positive for morphine
Source: PLoS One. 2017 Nov 16;12(11):e0187639. doi: 10.1371/journal.pone.0187639 (PMC5690676; doi:10.1371/journal.pone.0187639)
Supplement: S4 Table — (DOC) [file pone.0187639.s006.doc]

**S4 Table. Gene expression profiles for IP-10 and CLDN8 in tissues.**

|  | Transcripts per million (TPM) | |
| --- | --- | --- |
| Tissues | IP-10 | CLDN8 |
| Intestine | 4 | 38 |
| Kidney | 33 | 94 |
| Lung | 20 | 8 |
| Mammary gland | 33 | 39 |
| Prostate | 10 | 52 |

The information from NCBI UniGene (https://www.ncbi.nlm.nih.gov/unigene/)
